# Sterility and the X-Ray

**Published:** 1905-03

**Authors:** 


### STERILITY AND THE X-RAY.

It would have been amazing if there had not come up something more serious than burns to check what may be called the Roentgen rays in medicine. That something seems at last to have been encountered, at least if there is final truth in what has recently been put forward concerning the influence of the rays upon the sexual glands. They are said to destroy the power of the testicles to elaborate potent spermatozoids and that of the ovaries to produce ovules susceptible of fertilization. In other words, they blot out the reproductive power in an individual, male or female, whose sexual glands come under their influence. Whether this blasting effect is lasting or only temporary does not appear to have been settled as yet, even if we may set it down as absolutely established that it occurs at all in the great majority of cases.

At all events the impression that exposure of the testicles or ovaries to the action of the Roentgen rays is destructive of their procreative function has to a great extent taken possession of medical men, and it is sure to be spread to the laity. What is to be the result? As regards the testicles, probably it will be of little consequence. In the case of the ovaries, however, fear of injuring their power of ovulation, both on the part of the physician and on that of the patient, seems likely to interfere materially with the availability of the rays for diagnostic purposes so far as the pelvic contents in women are concerned. Moreover, this dread may work moral disaster, for we fear it can not be doubted that there is many a woman who would eagerly destroy within herself the potentiality of maternity if she thought she could accomplish her object by so simple an expedient as exposure to a subtle agency that would not give her pain or endanger her life or leave upon her a physical stigma that would lay her open to the suspicion of having resorted to such an unworthy device. On these accounts it is to be hoped that our present impression of the danger to fertility of exposing the sexual glands to the action of the Roentgen rays may prove to have been hastily formed.—*N. Y. Med. Jour. and Phila. Med. Jour.*

